# A methodology for evaluating digital contact tracing apps based on the COVID-19 experience

**DOI:** 10.1038/s41598-022-17024-2

**Published:** 2022-07-26

**Authors:** Enrique Hernández-Orallo, Pietro Manzoni, Carlos T. Calafate, Juan-Carlos Cano

**Affiliations:** Computer Engineering Department (DISCA), Universitat Politècnica de València, 46022 Valencia, Spain

**Keywords:** Computer science, Health policy, Epidemiology

## Abstract

Controlling the spreading of infectious diseases has been shown crucial in the COVID-19 pandemic. Traditional contact tracing is used to detect newly infected individuals by tracing their previous contacts, and by selectively checking and isolating any individuals likely to have been infected. Digital contact tracing with the utilisation of smartphones was contrived as a technological aid to improve this manual, slow and tedious process. Nevertheless, despite the high hopes raised when smartphone-based contact tracing apps were introduced as a measure to reduce the spread of the COVID-19, their efficiency has been moderately low. In this paper, we propose a methodology for evaluating digital contact tracing apps, based on an epidemic model, which will be used not only to evaluate the deployed Apps against the COVID-19 but also to determine how they can be improved for future pandemics. Firstly, the model confirms the moderate effectiveness of the deployed digital contact tracing, confirming the fact that it could not be used as the unique measure to fight against the COVID-19, and had to be combined with additional measures. Secondly, several improvements are proposed (and evaluated) to increase the efficiency of digital control tracing to become a more useful tool in the future.

## Introduction

Little did we know when the COVID-19 pandemic started that it would have such a huge impact on our society. Not only has it caused millions of deaths, but it has also changed our way of living. Fortunately, with the introduction of vaccines and new treatments, the pandemic is on the verge of being controlled.

The initial outbreaks of COVID-19 were tackled mainly by lockdowns and a stringent social distance to slow down the pandemic and make it more manageable by national health systems. After those initial outbreaks, countries started to use manual contact tracing, testing the symptomatic individuals, and when positive cases were detected, their prior contacts were traced and isolated. Thus, this contact tracing procedure, although being an arduous and slow manual task, is a more selective and effective isolation measure than general lockdowns^1^.

Nevertheless, the first studies have shown that asymptomatic individuals have caused about 80% of the infections^2^ since most infected individuals do not experience severe symptoms. This fact, combined with the large basic reproductive number of the COVID-19, implies that contact tracing has to be fast and accurate in order to be effective.

*Digital Contact Tracing* (DCT) was proposed with the aim of speeding up and extending this tracing contact process^3^. As its name suggests, Digital Contact Tracing relies on electronics tracking systems, mainly based on mobile devices, to determine risky contacts. Several contact tracing mobile apps were developed, such as Europe’s PEPP-PT^4^ and MIT’s SafePath^5^. Nevertheless, it was finally Apple and Google who teamed up in April 2020 to develop the most widely adopted solution, which was integrated into both Apple and Google mobile phone operating systems.

Digital Contact Tracing is based on the results of several years of research in Mobile Computing, particularly Opportunistic Networking (OppNet) and Mobile Crowdsensing. OppNet is based on the *opportunity* of exchanging messages between nearby devices when some type of direct and localised communication link is established (for example, through a Bluetooth or WiFi direct channel)^6^. The behaviour and dynamics of OppNets are similar to an epidemic spread of messages. Actually, many of the models used to evaluate these networks have been adapted from well-known epidemic population models^7^.

In a previous study^8^, we studied the efficiency of smartphone-based contact tracing, even before the first contact tracing apps were introduced. The main conclusions of that work are that digital contact tracing can only be effective in controlling an outbreak when the adoption ratio is very high (more than 80%); if this adoption ratio is even slightly lower (around 60%), it needs to be combined with other measures, although its effectiveness is moderate. Unfortunately, the real usage of these applications was around 20%, so, as we forecasted, their effectiveness was low.

The crisis and urgency provoked by the COVID-19 sped up the research and development of new methods to fight against this pandemic, being the fast development of ARNm vaccines the most outstanding example. Similarly, a usually lengthy process that requires researching, developing, testing, deploying, and finally evaluating an application such as digital contact tracing, was shortened to a few months. This experience allows us to evaluate the efficiency of these deployed applications in such a short time.

The main goal of this paper is to develop a systematic methodology based on an epidemic model to evaluate quantitively the effectiveness of current and future digital contact tracing apps. To this end, we firstly evaluate and analyse the main causes that can impact their efficiency and effectiveness. Several aspects are considered, such as adoption ratio, user’s willingness to check their exposure, tracing speed, and application model (centralised vs decentralised). Then, based on our evaluation methodology, we propose several changes to digital contact tracing to be more effective in future epidemics. In addition, our model allows evaluating the impact of these changes depending on the main characteristics of future epidemics and the additional measures taken. This way, by knowing the real impact of these technological decisions, we will be able to improve these applications to manage an epidemic effectively. Note that this paper is devoted only to evaluating the technical aspects of digital contact tracing and not the organisational, medical, sociological, and political aspects that impact the success of these applications.

The performed evaluation shows that the adoption ratio is the main factor that impacts the effectiveness of digital contact tracing. Only with high adoption ratios can an outbreak be controlled. Nevertheless, in most cases, this is not a realistic scenario. Therefore, digital contact tracing should be used as an add-on to standard epidemic mitigation measures, such as social distancing or manual contact tracing. This way, it can contribute to the reduction of cases while being more selective than general lockdowns.

The remainder of the paper is organised as follows: next, we overview the related work. Then, the methods of this article are introduced, particularly how digital contact tracing efficiency and effectiveness can be characterised based on the proposed deterministic model. Section evaluation and results, firstly evaluates the effectiveness of the deployed digital contract tracing apps by modelling a real scenario and then by studying how digital contact tracing can be improved for future epidemics. Finally, the paper ends with a discussion of the results and the main conclusions.

## Related work

Understanding the patterns of how a disease is spread, as well as the cause of these patterns, is essential to mitigate and prevent infections. This is the main goal of epidemiology. Recently, with the advent of Information and Communications Technology, a new term has been coined: *Digital epidemiology*. Digital epidemiology is simply epidemiology that uses digital data^9^. The goal is to predict, prevent and control epidemics using technologies such as internet-based surveillance, infectious disease modelling and simulation, remote sensing, telecommunications and mobile phones^10^. Among these technologies, mobile computing, particularly smartphones, has allowed users to share data, which is the basis of Mobile CrowdSensing. Mobile Crowdsensing is mainly based on two sensing approaches: mobile sensing, that is, gathering data from the mobile’s hardware sensors; and social sensing, which exploits data from social networks^11,12^.

Particularly, wireless sensor network technologies, such as ZigBee or Bluetooth, can be used to detect and trace contacts. Salathe et al.^13^ performed one of the first experiments using MOTES to detect contacts. Regarding mobile phones, the *FluPhone* application developed at Cambridge University^14^ is considered one of the first attempts in this area; in particular, it used Bluetooth for estimating physical contacts and asked users to report if they had flu-like symptoms.

In the initial stages of the COVID-19, several frameworks for implementing digital contact tracing were developed, such as the *Pan-European Privacy-Preserving Proximity Tracing* (PEPP-PT)^4^, and *SafePaths*^5,15^. Finally, Google and Apple have teamed up to develop and integrate similar solutions into their iOS and Android operating systems. This framework was widely used for implementing the different digital contact tracing apps deployed in countries and states.

Digital contact tracing is based on detecting risky contacts mainly using the communications technologies embedded in mobile phones. Nevertheless, this detection is far from being reliable since it depends primarily on the use of the received signal strength, which can vary depending not only on the distance but also on the orientation of the devices, the absorption level of the human body, and the location^16,17^. Finally, for further information about digital contact tracing technologies and their requirements, please refer to the following surveys^18–21^.

Traditional contact tracing has been widely used as it is a useful tool for controlling the spread of infectious diseases. The evaluation of the different contact tracing strategies has been a key research topic in epidemiology. The evaluation of contact tracing is based mainly on two modelling approaches^22^: *Population-based modelling* is a macroscopic approach, depicting disease dynamics on a system level; *Agent-based modelling* is a more microscopic approach dealing with each individual as an agent, modelling their movements and infection states. In general, although this method is more realistic, it can be computationally demanding.

In this paper, we follow the population-based modelling by developing an epidemic model, as do most of the previous evaluations^23^. These models are based on the basic SIR (Susceptible Infected Recovered) model, also considering quarantine, isolation measures, and incubation periods^24–27^. Eames et al.^1^ showed that contact tracing is effective only at the early stages of an outbreak and when the number of infection cases remains low. Particularly interesting are the results of the work by Farrahi et al.^28^, which evaluated digital contact tracing using population-based models.

With regard to the COVID-19, some studies have evaluated contact tracing. Ferretti et al.^3^ showed that traditional contact tracing in the first outbreak did not prevent the COVID-19 epidemic, mainly due to the high number of asymptomatic cases that remain undetected. Therefore, they proposed digital contact tracing as a way to speed up the detection process. Kretzschmar et al.^29^ obtained similar conclusions: reducing the testing delay is the most crucial aspect to enhance the effectiveness of contact tracing. Cencetti et al.^30^ showed that isolation and tracing alone are unlikely to be sufficient to keep an outbreak under control, and additional measures need to be implemented simultaneously. Hellewell et al.^27^ draw a similar conclusion through a simulated model, showing that highly effective contact tracing was enough to control a new outbreak of COVID-19 within three months, even when only 79% of the contacts were traced. Finally, Lambert^31^ has also proved, using an analytical model, that moderate rates of adoption of a digital contact tracing app can reduce the reproductive number $R_0$, but are by no means sufficient to reduce it below 1 (that is, to have control of the outbreak) unless other measures are taken.

Recently, several papers have studied and evaluated the effectiveness of digital contact tracing. Rodríguez et al.^32^ describe the results of the first experimental deployment of the Spanish digital contact tracing that took place in one of the Canary Islands in July 2020. With an adoption ratio of 33%, only 10% of all the potential cases were detected. Wymant et al.^33^ present the epidemiological results of the NHS COVID-19 app deployed in England and Wales. With an adoption ratio of 28%, their analysis suggests that a large number of cases of COVID-19 were averted by the NHS App, and estimated that for every percentage point increase in app uptake, the number of cases could have been reduced by 0.8%. Finally, Li et al.^34^ performed a survey to evaluate the reasons behind the low adoption rates of the COVID-19 contact-tracing apps. Grekousis and Liu^35^ studied the real impact of several applications on reducing the effective reproductive number.

Moreno et al.^36^ evaluate several factors that have impacted the effectiveness of digital contact tracing, such as the role of age, transmission setting, detection, and household isolation using a compartmental model. The evaluation of actual contact tracing apps by Maccari et al.^37^ is quite pessimistic: they claimed that there is no scientific evidence of their efficacy in slowing down the spread of the virus, and propose to re-think some privacy-invasive measures that have made digital contact tracing inefficient. Other studies evaluated aspects such as privacy and deployment policies that have impacted on their adoption rate and consequently on the effectiveness of digital contact tracing^38–40^. A more holistic approach is followed by Shubina et al.^41^, using a wide range of factors: technical, epidemiological and social ones that are incorporated into a compact mathematical model for evaluating the effectiveness of digital contact tracing solutions.

Summing up, most of the previous papers were focused on the epidemiological aspects of the COVID-19 and contact tracing effectiveness. Instead, our paper aims to provide a quantitative methodology to assess the efficiency of current and future digital contact tracing apps, focusing on those technological aspects that have a significant impact. This way, knowing the real impact of technological decisions will allow us to improve these applications in the near future.

## Methods

Generally speaking, contact tracing is the procedure of identifying individuals who may have come into contact with an infected individual. Then, by tracing back the contacts of these infected people, public health professionals aim to reduce infections in the population, applying measures such as selective isolation. Traditional manual contact tracing is a laborious and lengthy task involving personal interviews that, in most cases, provide only vague information about the previous contacts. Consequently, digital contact tracing aims to automate this task, mainly by using users’ smartphones to detect contacts between infected and susceptible people, and trace back contacts.

We start this section by detailing how a risky contact can be determined and the architecture of digital contact tracing, focusing on those aspects that will impact its efficiency. Then we characterise this efficiency which will be used in the proposed model for assessing digital contact tracing. We differentiate between the terms efficiency and effectiveness. Effectiveness is used more as a medical term, that is, how well a treatment works when people are using it, and it can be measured as the number of infected or dead people averted with digital contact tracing. Efficiency is used more as a technological term and allows scientists to evaluate how well digital contact tracing is working, for example, by reducing the number of false alerts or by increasing the tracing speed.

### Risk contacts estimation

One of the critical aspects of digital contact tracing is how to estimate risk contacts using the underlying technology of current users’ smartphones. From the beginning of the COVID-19, health authorities have considered a risk contact someone who is in close contact (less than two meters away for at least 15 min) with a person who tested positive^42–44^.

Current smartphones can provide several ways to determine these close contacts using localisation and communication technologies, such as GPS, Wi-Fi, Bluetooth, beacons, or even QR codes. The final goal is to provide a method to detect risky contacts with enough precision for contact tracing.

As most COVID-19 applications finally used Bluetooth for its greater precision and privacy, we briefly summarise this detection technology. When two Bluetooth devices communicate, the sender emits its signal at a certain power level, while the receiver observes this signal at an attenuated power level known as the received signal strength indicator (RSSI). Since attenuation increases with the square of the distance, the distance between two Bluetooth devices can be inferred using a Path Loss Model. On the other hand, the duration of a contact is estimated by periodically sending Bluetooth messages and calculating their distance. However, RSSI values typically fluctuate in time or are influenced by other factors such as obstacles and reflections^45^. Several studies have shown that it depends on factors such as the relative orientation of handsets, absorption by the human body, type of devices used, and if the contact is indoor or outdoor^17,46^. For example, the evaluation shown in^46^, in which several distances and scenarios were used using the Android Beacon Lib., showed an accuracy of close to 50% indoors and of about 70% outdoors.

Nevertheless, this is only referred to the accuracy of estimating if a contact is in the 2 meters range, not about the real exposure to the virus. The risk also depends on many other factors, such as the exposure intensity to the virus, the quality of the medium, and the susceptibility of the non-infected person.

A way to improve this precision is to use other smartphone sensors to detect the kind of location (indoor/outdoor) and the quality of the medium (temperature, sunny/cloudy). Considering this new information, and with the combination of machine learning techniques, it is possible to improve the accuracy to 83% indoors and 91% outdoors^45^.

**Figure 1:**
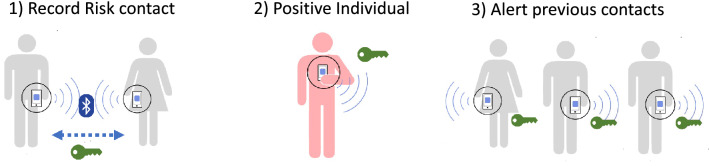
Digital contact tracing: (**1**) when the smartphones of two users are in range, they exchange their anonymous key codes; (**2**) if an individual is detected positive, it starts a process for tracing and identifying the previous contacts; (**3**) users are notified about a risky contact.

### Digital contact tracing architecture

Digital Contact Tracing works in a similar way to traditional contact tracing but uses smartphones to detect and record the possible risky contacts (see Fig. 1). The first step is to install on the user’s smartphone an app that will be active to monitor these contacts. When the users’ phones are in contact for at least 15 min and at a distance of less than two meters, the app understands that there has been a risky contact. To preserve privacy, the smartphones exchange anonymous key codes, which can be used to determine the identity of the people contacted. If a user is diagnosed as positive after performing a test, the app should be notified in order to start the process of tracking the user’s previous contacts, which will use the generated keys of their previous contacts to identify the users at risk. Then, users who have had a contact with the positive user will receive an alert.

Nevertheless, there have been several considerations for the design of these contact tracing apps, such as where the keys are stored, how the matching is done, some privacy issues, and the adoption requirements. Regarding where the keys are stored and managed, there are two different models: in the *centralised* approach, the generated keys are stored and managed in a central server. This way, when positives are detected, the users notify the application of their new status, which is transmitted to the central servers. Then, the centralised servers check for all her/his previous risk contacts, who are notified immediately by the application. On the contrary, in the *decentralised* approach, the generated keys are stored on the user devices. Only when a user is detected positive, the mobile application will upload the recent locally stored keys to the server, which will be distributed among all the users in order to match locally if they have been in contact with this individual. Although the decentralised approach seems to preserve the privacy of the users, it depends on their willingness to check and inform health authorities of this possible risky contact, being less effective than the centralised approach.

In both models, we consider the matching of keys and notifications to users to be completely automatic and immediate in order to shorten the tracing process. Finally, note that the centralised approach allows several variations. If required, the health authorities can have direct oversight of user data, so they can check, notify and manage previous contacts. It can also be used in combination with manual contact tracing, so the positives detected are notified to the tracing teams (the user is not notified automatically by the app), and the notifications to users could be delayed.

Another implementation issue is the adoption requirements of the app. That is, how and when the contact tracing app is activated. As the effectiveness of digital contact tracing depends on the adoption rate, this is a key aspect. There are different strategies for activating the app: mandatory use, opt-in and opt-out. A mandatory adoption implies that the application is always active, for example, by compelling their citizens to install the application and use it. This mandatory adoption may be viewed as a privacy violation in most countries. Therefore, most of the offered applications implemented the *opt-in* strategy for activating the app: users should download the app and proactively *opt-in* for using it, penalising its utilisation. Finally, the opt-out strategy assumes that the application is installed and activated by default (for example, by making use of an operating system update). Nevertheless, although the user still has the option to disable the application, most people would not opt-out^47^, and this will increase its utilisation.

The previous adoption requirements consider the entire population of a country or state, which makes high adoption ratios very difficult. Nevertheless, we can improve the utilisation and effectiveness if we consider only the people at specific locations, such as factories, music festivals, university campuses, retirement homes, and conferences (these groups are medically referred to as cohorts). The app should only work in those locations (for example, by using the GPS and establishing the tracing area where the smartphone can detect these contacts) and would be mandatory (no privacy issues can be raised as the app only traces the contacts in those locations). Additionally, for retirement homes, we can consider that the elderlies’ could use other more manageable devices, such as wristbands or necklaces, with detection capabilities similar to those of smartphones. Thus, considering the individuals in those cohorts, the adoption ratio could reach 100%.

Finally, and regarding the implementation decisions of digital contact tracing for COVID-19, the majority of countries chose the Google/Apple Exposure Notification API as the framework for implementing their apps^39^. This framework implemented a decentralised approach and Opt-in activation, limiting, as we will see, its efficiency. Other countries, such as China and South Korea, developed their own framework and application, a mandatory app with a centralised model.

### Characterising digital contact tracing efficiency

As detailed in the previous subsections, several technical aspects of digital contact tracing can have a huge impact on its efficiency: the precision of detecting risky contacts, some implementation decisions such as the centralised vs. decentralised approach, and the adoption model. Therefore, in this subsection, we first evaluate and parametrise the impact of these technical aspects; then, we introduce some simple expressions to evaluate the efficiency of contact tracing.

#### Parametrising digital contact tracing

Accuracy is fundamental in detecting real risky physical contacts, that is, a true positive contact. Nevertheless, as detailed in section "Risk contacts estimation", current smartphone risky contact detection is not precise enough. Smartphone-based detection can generate false negatives (a true positive contact is missed) and false positives (a false contact wrongly detected as positive).

To characterise the false and negative contacts, we use the following ratios: the True Positive Ratio (or sensitivity), $TPR$, is the ratio between the number of the detected positive contacts and the real number of positive contacts, and the False Positive Ratio, $FPR$ is the ratio between the number of negative contacts wrongly categorised as positive and the total number of actual negative contacts. The impact of these ratios in the contact tracing process is quite different: a greater $TPR$ implies that more infected individuals can be detected and isolated, and $FPR$ increases the number of people wrongly considered infected (i.e. a false alarm), and thus the people isolated unnecessarily.

For example, the first evaluation of England’s contact tracing app performed in August 2020 (based on version 1.4 of the Google/Apple Exposure Notification) showed a $TPR$ of 69% and a $FPR$ of 45%^48^. These numbers were not good, especially considering the high rate of false alarms that were generated, which undermined the people’s confidence in the app. A posterior refinement on the classification algorithm reduced this $FPR$ significantly.

The centralised and decentralised approaches have an impact on the contact tracing time and the tracing coverage. The contact tracing time *TT* is the time in days required since an individual is tested positive until the notification of his/her traced contacts. The tracing coverage *TC* is the proportion of previous contacts traced.

If we consider that in the centralised approach, all the keys are uploaded to a centralised server, when individuals test positive health authorities can immediately start the process of matching their previous contacts obtaining full tracing coverage (thus, $TT \le 1$ day and $TC=1$). On the contrary, this process is not as fast in a decentralised approach and depends on the users’ willingness. Firstly, when an individual is tested positive, she/he should notify the application. Secondly, as the matching is done locally, the potential previous contacts of this new positive individual should check the App. This checking will produce delays of several days in the notification ($TT>1$ days), and that some potential contacts are not notified, reducing the tracing coverage ($TC<1$). This last value will depend on the users’ willingness to check their App.

The adoption ratio has been shown to be a critical issue in the efficiency of digital contact tracing. The key question here is how many people are going to use the application. As detailed in the previous subsection, this rate depends on the adoption model. It is clear that a mandatory model will imply a high utilisation rate, while an Opt-in model will reduce its use significantly. Unfortunately, for the Opt-in model used in most countries, the utilisation rates were in the range of [0.15, 0.35], far below the necessary utilisation rates recommended for the models to be effective.

This adoption ratio (*AR*) can be used to estimate the number of contacts that can be traced, and, in some way, determine roughly the efficiency of the process as the proportion of the contacts detected to all real contacts. Note that, for detecting a contact, it is required that both individuals use the App. Therefore, the likelihood of detecting a contact is $AR \times AR$, which is the probability that in a real contact both individuals use the App. This probability means that the ratio of contacts detected is $AR^2$, which implies that a high adoption ratio is required in order to capture a considerable number of contacts (for example, with an adoption ratio of 0.25, only 6.25% of the contacts can be captured).

#### Measuring tracing efficiency

A simple way to measure the efficiency of contact tracing is to determine how many risky contacts can be detected. The first expression, the *true traced contacts ratio* $c_T$, determines the ratio of true risky contacts detected to all real risky contacts. This ratio can be obtained by taking into consideration the true positive ratio $TPR$, the adoption ratio *AR*, and the tracing coverage *TC*. All these parameters reduce the final number of detected contacts as follows:

1 $$\begin{aligned} c_T = TPR \cdot (AR)^2 \cdot TC \end{aligned}$$

A similar expression can be obtained to determine the ratio of false positives generated, or *false alerts ratio*, $c_A$, using the false positive ratio:

2 $$\begin{aligned} c_A = FPR \cdot (AR)^2 \cdot TC \end{aligned}$$

As an example, we can estimate the efficiency of England’s digital contact tracing app. The parameters have been obtained from^48^, and^33^: the true and false positive ratios were 69% and 45%, respectively; the adoption ratio was 28% (from a total population of 58.9 million), and the tracing coverage of 80%, estimated as the reported adherence to quarantine rules of the individuals who used the app. With these values, the true traced-contacts ratio $c_T$ was 0.0433, and the false alerts ratio $c_A$ was 0.0282. Regarding $c_T$, this means that only 4.3% of the real contacts were detected using the app, which can be considered a small efficiency. Nevertheless, in order to evaluate the real impact of these parameters when dealing with the COVID-19, we need to use an epidemic model.

**Table 1:** Notation table.

Symbol	Definition
*N*	Population
*S*, *I*, *R*	Susceptible, Infected and Recovered individuals classes
$Q_S$	Susceptibles in quarantine by tracing
$Q_I$	Infected detected and quarantined.
$Q_T$	Infected detected and being traced.
*V*	Vaccinated individuals
*v*	weighted efficacy of the vaccines
$R_0, R_e$	Basic and effective reproductive ratios ($R_0=kb/\gamma$)
*K*	*K*(*t*), contact per day and individual depending on time
*B*	*B*(*t*), probability of disease transmission depending on time
$\beta$	Transmission rate ($\beta =k \cdot b$)
$\gamma$	Recovery rate ($1/\gamma$ = days to recover)
$\delta$	Detection rate of infected individuals
$1/\tau _Q$	Average quarantine time
$1/\tau ^r_Q$	Average quarantine time minus $1/\tau _T$
$1/\tau _T$	Average tracing time.
*TT*	Contact tracing time. $TT=1/\tau _T$
$\Omega$	$\Omega (t)$,vaccination rate (per day) depending on time
$TPR$	True positive ratio
$FPR$	False positive ratio
*AR*	Adoption ratio
*TC*	Tracing coverage
$c_T$	True traced contacts ratio
$c_A$	False alerts ratio
IFR	Infection Fatality Rate.

### A model for assessing digital contact tracing

The model presented here is a Susceptible, Infected, Recovered (SIR) deterministic epidemic model, which considers not only the impact of the digital contact tracing technology through the parameters described in the previous subsection but also the effect of the quarantine measures taken in case an individual is detected positive, and the immunity due to vaccines. The goal is to obtain a model that reproduces the spread dynamics of the COVID-19 disease that will be used to evaluate the effectiveness of digital contact tracing.

The model we introduce here is a derivation of the stochastic epidemic model presented in our previous work^8^, in which we considered the heterogeneity of the contacts. Thus, in our new model, we consider a population of *N* individuals and homogeneity of the contacts. This new model also considers the effect of the temporal measures taken (such as social distancing and mask), along with the vaccination rates.

**Table 2:** Some estimated parameters for the initial variants (year 2020) of COVID-19^2,3,27,36^. The time unit is days. The column *value* shows the values used in our experiments. Note that some of these parameters can vary depending on the country and age group, as shown in the column *range*. Later variants, such as the extremely contagious Omicron, have different parameters.

Parameter	Value	Range
$R_0$	3	[1.5,6]
$\beta$	0.52	[0.25,1]
$\gamma$	1/10	[1/15,1/5]
$\tau _Q$	1/14	[1/20,1/10]
$\delta$	0.01	[0.002,0.2]
IFR	1%	[0.01,10]
v	0.8	[0.6,0.95]

**Figure 2:**
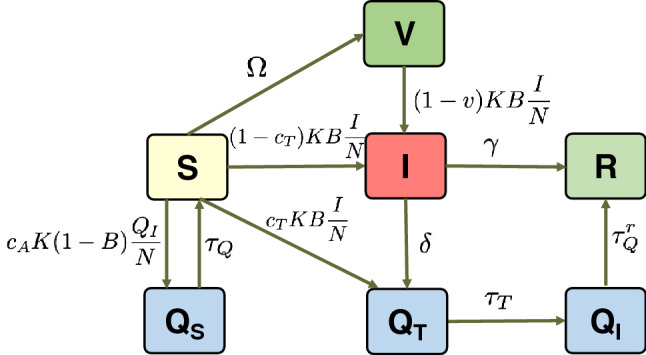
Transitions of our epidemic model and their rates.

#### Epidemic model

Epidemic models are usually based on the transmission rate (or risk) $\beta$, the rate at which an infection can be transmitted from an infected individual to a susceptible one. This rate can be obtained as the product of the average number of contacts with infected individuals during a day, *k*, and the transmission probability of the disease, *b*, where the time unit *t* is in days. Infected individuals recover after $1/\gamma$ days, where $\gamma$ is known as the recovery rate. These values are related to the basic reproductive ratio as $R_0=kb/\gamma$. $R_0$ represents the expected number of new cases directly generated by a single case. When $R_0 > 1$, the infection will start spreading in a population, but not if $R_0 < 1$. Generally speaking, the larger the value of $R_0$, the harder it is to control the epidemic. When measures are taken, this reproductive ratio can be reduced, and it is usually referred to as the effective reproductive number $R_e$. For the COVID-19, in Table 2 we can see the estimated parameters of its transmission. These parameters are estimated when no health measures are taken. As for COVID-19, we have experienced that when temporarily applying physical measures such as social distancing and wearing masks, both the probability of transmission and the number of contacts were reduced. Therefore, in this model, we consider the time dependency of these parameters to model the effect of these temporal measures: *B*(*t*) and *K*(*t*). Note that, for simplicity of notation, we will omit the time in the expressions that follow. The number of casualties can be obtained from the whole number of infected individuals multiplied by the Infection Fatality Rate (IFR).

Vaccination reduces the probability of infection and its transmission drastically, and thus the mortality rates. Fortunately, for the COVID-19, it has been the definitive solution. Nevertheless, vaccines are not 100% effective, so vaccinated people can get infected. This effectiveness depends on the type of vaccine (for example, the effectiveness of Pfizer’s vaccine is around 95% and the AstraZeneca’s one around 70%.). In our model, we take into account the weighted average effectiveness of the vaccines used in a country (*v*). We also consider a vaccination rate (per day) depending on time $\Omega (t)$ to model when the vaccines were introduced and their rate.

As detailed in the previous subsection, digital contact tracing cannot trace all real contacts positively, and it can even generate false positives. We obtained two expressions for measuring this efficiency: the true traced contacts ratio $c_T$ (Eq. 2), and the false alerts ratio $c_A$ (Eq. 1). Nevertheless, if tracing time is greater than one, that is, for the decentralised schemes, the $c_T$ and $c_A$ ratios need to be normalised considering the tracing time *TT*, since it will take more time to trace the previous contacts. Thus, the contacts are distributed among the days that last the tracing process, in the following way: $c_T^n=q/(1/\tau _T)=c_T\tau _T$ and $c_A^n=c_A\tau _T$.

In our model, we assume that a newly detected infected individual is immediately isolated, and his/her previous contacts are evaluated using digital contact tracing. Then, these previous contacts are considered to be quarantined. Therefore, besides the common SIR classes (*S*, susceptible individuals; *I*, infected individuals; *R*, individual recovered;) we define three new classes for the individuals being in quarantine. Namely, $Q_I$ refers to an infected individual that has been detected (or traced) and therefore quarantined; $Q_S$ to a susceptible individual that is quarantined after being traced; and $Q_T$ to an infected individual that has been detected and is being traced. There is also a class *V* for the vaccinated people. Finally, refer to Table 1 for the notation used in the model.

The transitions between classes and their rates are depicted in Fig. 2. The time unit is one day, as most human epidemic models do. The general transition rate from susceptible to infected is $KB\frac{I}{N}$, which depends on the transmission rate of the disease, *B*, the average number of contacts, *K*, and the ratio of infected individuals to the population, $\frac{I}{N}$. Nevertheless, in our model, we distinguish between the infected individuals that have been traced positive and the rest. The transition $S \rightarrow I$ occurs when a susceptible individual that has not been traced positive gets infected. Thus, the previous general transition rate is multiplied by $(1-c_T)$, which is the ratio of non-traced contacts. Therefore, class *I* contains infected people who have not been detected positive and are not quarantined. The transition $S \rightarrow Q_T$ is for the susceptible ones that are infected and are detected positive (a true positive), mainly using digital contact tracing (which is why this transition rate is multiplied by $c_T$). Note that infected individuals that are in class *I* can be also detected by tests (PCRs) with a $\delta$ rate, traced back, and quarantined (transition $I\rightarrow Q_T$).

Individuals stay in quarantine for a total of $1/\tau _Q$ days. Nevertheless, we divide this quarantine into two phases. The first phase (transition $Q_T \rightarrow Q_I$) is the time needed to trace their previous contacts, which is the tracing time $TT=1/\tau _T$. This phase is added to evaluate the impact of this time, for example, to consider the delay incurred in the decentralised approaches. After this tracing time, the infected individuals stay at class $Q_I$ for the rest of the quarantine, $1/\tau ^r_Q$ = $1/\tau _Q- 1/\tau _T$, and finally recover (transition $Q_I \rightarrow R$). Finally, transition $I \rightarrow R$ represents the individuals who remain undetected and recover from the disease, with a recovery rate $\gamma$. Note that this case also includes asymptomatic individuals.

Now we consider the effect of false alarms ($c_A$). The effect of false alarms (false positives) is that some non-infected individuals will be considered as infected and, therefore, wrongly quarantined. This corresponds to the transition $S \rightarrow Q_S$, which considers the probability of not transmitting the disease $(1-b)$, and the ratio of false alarms generated, $c_A$. Class $Q_S$ is introduced to evaluate the individuals that are *unnecessarily* quarantined. When the quarantine ends (for $1/\tau _Q$ days), these individuals return to the susceptible class (transition $Q_S \rightarrow S$).

Finally, transition $S \rightarrow V$ occurs when a susceptible individual gets vaccinated with rate $\Omega$. Nonetheless, some of the vaccinated people could get infected. This is represented by transition $V \rightarrow I$, with a rate of $(1-v)KB\frac{I}{N}$, that depends on the weighted efficacy of the vaccines. In order to simplify the model, we do not consider that the vaccinated people are traced and quarantined. Note that, as a model, we have simplified or omitted some transitions with the aim of making the model amenable while keeping the fundamental behaviour that will help us to evaluate digital contact tracing.

From these transitions and rates, the epidemic model is defined as follows:

3 $$\begin{aligned} \begin{aligned} S'&= -(1-c_T)KB \frac{I}{N} S- c_T KB\frac{I}{N} S - c_A K(1-B) \frac{Q_I}{N} S \\&\quad -\Omega S + \tau _Q Q_S \\ I'&= (1-c_T)KB\frac{I}{N} S + (1-v)KB\frac{I}{N} V - \delta I - \gamma I \\ R'&= \gamma I + \tau _Q Q_I \\ Q_S'&= c_A K(1-B)\frac{Q_I}{N} S - \tau _Q Q_S \\ Q_I'&= \tau _T Q_T -\tau ^r_Q Q_I \\ Q_T'&= \delta I + c_TKB\frac{I}{N} S -\tau _T Q_T \\ V'&= \Omega S - (1-v)KB\frac{I}{N} V \end{aligned} \end{aligned}$$

Note that, for simplicity of notation, the time has been omitted in all the classes, and in the *K* and *B* functions. For example, for class *S*, $S'=dI(S)/dt$ and $S=S(t)$). This model is solved numerically, considering an initial value for *I*, *R* and *S* classes so $S(0)=N-R(0)-I(0)$, and the other classes are set to zero. The model can be solved for a given time (for example, one year), or until the infection is over.

#### Assessing the effectiveness of digital contact tracing

The effectiveness of digital contact tracing can be assessed in several ways. The highest level of effectiveness would be when it could control an outbreak, that is, when the number of infected individuals decreases. Considering the Eq. (3) of the epidemic model, we can determine this condition when $I'$ is negative as:

4 $$\begin{aligned} \left( \frac{(1-c_T)S}{N} + \frac{(1-v)V}{N}\right) R_e\gamma < \delta + \gamma \end{aligned}$$

considering that $KB=R_e\gamma$. We prefer to use the $R_e$ number as it is a more simple (and known) figure to express the intensity of an epidemic. If we analyse this expression, we can determine the main components that can lead to the control of an outbreak. The term $\frac{(1-c_T)S}{N}$ is the proportion of susceptible people that can be infected without being detected and quarantined. Similarly, the term $\frac{(1-v)V}{N}$ is the ratio of vaccinated people that can be infected. If we substitute these terms in 4 by $S_{RN}$ and $V_{RN}$ we have:

5 $$\begin{aligned} (S_{RN} + V_{RN})R_e\gamma < \delta + \gamma \end{aligned}$$

Particularly, we can see that, in order to control an outbreak, we should reduce component $S_{RN}$ by improving the efficiency of contact tracing ($c_T$) or by reducing the number of susceptible individuals, that is, reduce component $V_{RN}$ by improving the efficacy of the vaccines (*v*), reduce the transmission rate ($R_e$) or, alternatively, increase the detection ratio ($\delta$).

Other important figures to assess the effectiveness are the whole number of infected individuals and deaths. These values can be obtained by solving the model for a given time or until the infection is over ($I<1$). Then, we obtain numerically the number of accumulated individuals infected over the evaluated period, considering the individuals who move from classes S to I. Note that we can also obtain the number of deaths by multiplying the whole number of infected people by the Infection Fatality Rate (*IFR*). Nevertheless, reducing these values (infected individuals) can imply the application of severe measures such as quarantines. Thus, we get the accumulated number of people quarantined $Q_a$, which is obtained as the number of individuals that transition to classes ($Q_S$,$Q_I$ and $Q_T$). A highly effective contact tracing based quarantine will minimise the number of people quarantined while controlling the spread of the disease.

Finally, we can also evaluate the impact of false alerts ($c_A$). As described in the model, less precise contact tracing increases the number of susceptible quarantined individuals $Q_S$, that is, the individuals that are wrongly detected and quarantined. So, we can count the individuals that transition into class $Q_S$ as the number of generated false alerts.

## Evaluation and results

This section has a twofold objective. Firstly, we evaluate the effectiveness of some deployed digital contact tracing apps under scenarios that resemble the ones generated by the COVID-19. The goal is to evaluate why digital contact tracing has not been as effective as expected. Secondly, we study and evaluate the conditions and configurations that could make it more effective in the future.

**Figure 3:**
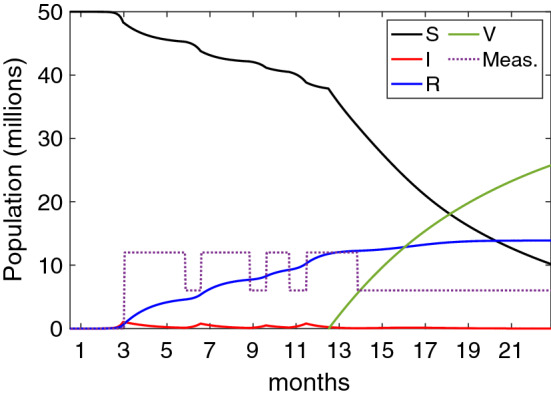
Real scenario for digital contact tracing showing the epidemic dynamics. Note the measures (*meas.*) line represents three levels of intensity, and it is not related to the x-axis.

### Effectiveness of deployed digital contact tracing

In this section, we use our proposed methodology to evaluate some of the real contact tracing Apps used to fight against the COVID-19. Particularly, we firstly evaluate a decentralised approach such as England’s NHS COVID-19 App, which is based on the Google/Apple API. Secondly, we evaluate a centralised approach, such as those deployed in China (Health Code) and South Korea (Self-quarantine Safety Protection App).

In order to evaluate the effectiveness of the deployed digital contact tracing apps, we consider a scenario that resembles the ones caused by the COVID-19. Since the beginning, in the first month of 2020, the COVID-19 generated several waves that have been mitigated by imposing different measures to reduce its diffusion. At the beginning of 2021, most countries started with vaccination, which seems to be the final solution to this pandemic. This scenario has been reproduced using our epidemic model, as shown in Fig. 3. We consider a population of 50 millions, an initial outbreak with 10 infected individuals (that is, $I(0)=10$), and the COVID-19 parameters shown in Table 2.

Specifically, we have modelled four waves with their subsequent measures, represented with three different intensity levels in the graph. The high level represents stringent measures such as a general lockdown; the medium level, social distancing and wearing masks; and finally, no measures (only at the beginning, when health authorities were not aware of the risk of the CIVID-19 pandemic). In our modelled scenario, we can see the effect of the stringent measures, which reduced the effective reproductive ratio $R_e$ to values around or below one, and so did the spread of the virus. Finally, we can see the impact of vaccination, which reduced the spread of the virus, evidencing that herd immunity was near. Note that the vaccinated class (*V*) contains only the individuals who were vaccinated and had not been infected. The individuals in the recovered class (*R*) could have received the vaccine. At the end of the evaluated period, the total number of infected people was 12.7 million with a death toll of 127.000, which are coherent with the real figures of some European countries such as the UK, France, Italy and Spain.

Using this scenario, we evaluate the impact of digital contact tracing considering the most deployed platform: the one based on the Google/Apple API, which is a Bluetooth-based, decentralised approach with Opt-in activation. Particularly, we focus our study on England’s digital contact tracing App (see the end of section "Measuring tracing efficiency" for more details). Thus, we consider the following parameters: true positive ratio $$ TPR =0.69$$, false positive ratio $$ FPR =0.45$$, adoption ratio $$AR=0.28$$ and tracing coverage $$TC=0.8$$. We also consider a distributed approach, so the average tracing time is two days ($$1/\tau _T=2$$).

**Figure 4:**
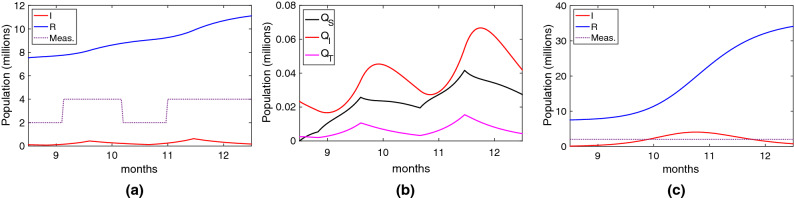
Effectiveness of England’s NHS contact tracing App based on Google/Apple API between September to December 2020. In these figures, the susceptible individuals are not plotted. (**a**) Infected and recovered individuals under the real scenario; (**b**) People quarantined; (**c**) Infected and recovered individuals with no stringent measures taken.

The first outcome is that digital contact tracing was not able to control any outbreak with the configuration and performance of England’s App. Considering the threshold for controlling a COVID-19 outbreak determined by Eq. (5), we find that it is far from controlling the epidemic. Satisfying this condition would require an adoption ratio greater than 80% with a true positive ratio of 0.8, clearly an unrealistic goal. Considering the application of mild measures that could reduce the value of $R_e$ to values around 1.5, the utilisation required should have been 65%, which is the value determined by the first models^3,8,27^.

Thus, discarded the outbreak control, we evaluated the effectiveness of digital contact tracing by determining the number of cases averted (infected individuals and deaths). We restricted our evaluation to the last four months of 2020 when digital contact tracing was initially introduced and used. Although in some countries these apps were active for several months more, the fact is that factors like starting the vaccination process, and a loss of confidence in them, caused their usage to decrease rapidly.

The results are shown in Fig. 4a. Compared to the results without digital contact tracing, the reduction of infected individuals is relatively small. During this period, the total of infected people was 3.57 million, compared to the 4.16 million if digital contact tracing was not considered. This means that approximately 0.59 million infected cases and 5900 deaths were averted. It goes without saying that every life saved counts, but more was expected. It is worth standing out that these results are quite similar to the ones presented by by Wymant et al.^33^, which evaluated the real effectiveness of the England NHS COVID-19 App for a similar period. These results were the consequence of applying selective quarantines, which is a very draconian (and annoying) measure. We can see the different types of quarantine in Fig. 4b, with an accumulated number of quarantined individuals ($Q_a$) of 1.89 million.

One of the causes of the low adoption ratio was the impact of false alerts, which undermined people’s confidence in digital contact tracing. This is reflected by the $Q_S$ line in Fig. 4b, which is quite high due to the moderate-high false positive ratio (0.45). The total of individuals wrongly quarantined by false alarms is 0.6 million, justifying people’s loss of confidence in the application.

Note that the previous evaluation considers that some stringent measures were taken during the use of digital contact tracing. What would have happened if these measures had not been taken? We can see the results in Fig. 4c, where most individuals would get infected (around 35 million), and the death toll would be 350.000 individuals.

**Figure 5:**
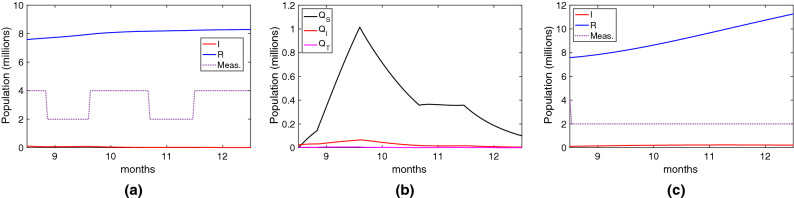
Effectiveness of the Chinese/Korean tracing applications between Sep-Dic 2020. In these figures, the susceptible individuals are not plotted. (**a**) Infected and recovered individuals under the real scenario; (**b**) People quarantined; (**c**) Infected and recovered individuals with no stringent measures taken.

The previous results have evidenced the limited effectiveness of the Google/Apple API based application (and particularly, the English NHS App). Now, we consider the App deployed in China (Health Code)^49^ and South Korea (Self-quarantine Safety Protection App)^50^. These applications were mandatory (so we consider an adoption rate of 80%) and used the centralised approach hence achieving full tracing coverage ($$TT=1$$ AND $$TC=1$$). The detection of contacts was based on GPS and assisted with QR codes, which gives a smaller precision when compared to Bluetooth (^51^): true positive ratio $$ TPR =0.5$$ and false positive ratio $$ FPR =0.4$$. Using these values, we repeated the same experiments and scenarios of the Google/Apple App shown in Fig. 4.

The results are shown in Fig. 5c. Firstly, we can see in Fig. 5a that the use of the Chinese/Korean digital tracing application combined with different measures significantly reduces the number of infected people. During this period, the total of infected people is reduced to 0.7 million (compared to the 3.57 million of the Google/Apple app.). This reduction has a significant effort, with a significant increase in the number of people quarantined, as shown in Fig. 5b. These results clearly reflect what happened in those countries, the infection was controlled better by imposing stringent quarantines. Finally, we evaluate the case when no other measures are taken. The results, shown in Fig. 5c, evidence that the outbreak cannot be controlled. Nevertheless, when compared to the ones in Fig. 4c, there is a significant reduction in the number of people infected.

Summing up, we have assessed the effectiveness of two different types of digital contact tracing apps. The ones based on the Google/Apple API were quite ineffective when compared to the Chinese/Korean ones. This improved effectivity is not only due to their high (mandatory) adaption rate but also due to their centralised architecture. Nevertheless, their low precision imposes a very high rate of (unnecessary) quarantines, so their efficiency is very low.

### Controlling a future epidemic

According to the previous evaluation, we have confirmed the relatively low performance of digital contact tracing in the COVID-19 real scenarios and the impossibility of controlling an outbreak. In this subsection, we study under which conditions a future virus outbreak could be controlled using Eq. (4). We do not know the intensity of a future epidemic, so this evaluation will depend on its reproductive ratio ($R_e$). For example, some very common infectious diseases have very high $R_0$ values: rubella, 6–7; chickenpox, 10–12; measles, 12–16.

**Figure 6:**
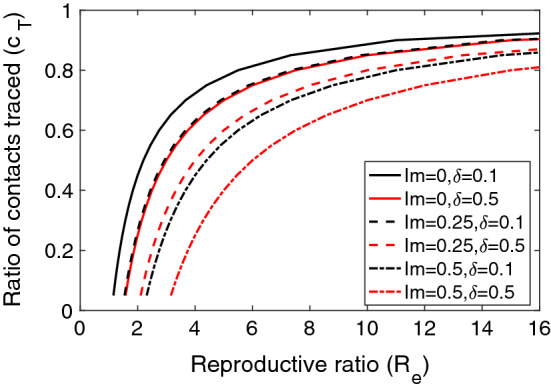
Digital contact tracing thresholds for controlling an outbreak based on Eq. (4), considering different values of the ratio of immunised individuals (Im) and detection rate ($\delta$). The pair of values above the lines results in a disease-free equilibrium.

The efficiency of digital contact tracing can be measured by the ratio of contacts traced ($c_T$). Additionally, other factors can reduce this required efficiency: the ratio of immunised individuals, which can comprise both the recovered individuals and the vaccinated ones, and the detection rate. The threshold plot is shown in Fig. 6, where the areas above the lines represent the values of $c_T$ necessary for controlling an outbreak depending on its reproductive ratio $R_e$. Additionally, we plot several curves for different values of the ratio of immunised individuals and detection rate. As expected, we can clearly see that the greater the reproductive ratio, the greater the required ratio of contacts traced, and thus a greater efficiency is needed. This required efficiency can be lowered if the detection ratio is high or with the ratio of immunised people.

For example, for COVID-19, with a reproductive ratio $R_e=3$ and no immunised individuals, the required value of $c_T$ would be greater than 0.65, which is a very high value. Recall from section "Measuring tracing efficiency" that the England’s digital contact tracing $c_T$ values was 0.043. As shown in the figure, the required efficiency will depend on the intensity of a future epidemic. For example, with reproductive ratios below 2, and considering a high detection ratio, the required ratio of contacts traced will be around 0.3, which is still a high figure.

Summing up, it is essential to increase the ratio of contacts traced, and as detailed in expression 1 mainly by increasing the adoption ratio and also by obtaining a higher tracing coverage and true positive ratio.

### How to improve the efficiency

**Figure 7:**
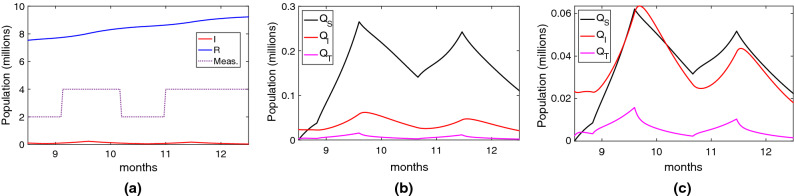
Efficiency of the contact tracing application considering an adoption ratio of 0.7. (**a**) Infected and recovered individuals under the real scenario; (**b**) People quarantined; (**c**) People quarantined when the precision is increased to $$ FPR =0.1$$ and $$ TPR =0.8$$.

In this subsection, we study how to improve digital contact tracing efficiency. It is clear that one of the main factors that affect efficiency is the adoption ratio. Considering the same scenario as in Fig. 4, if we increase the adoption ratio to 0.7, which returns a value of $c_T$ of 0.27, we can see in Fig. 7a that the reduction of infected individuals is quite significant (now 1.69 million), but at the cost of increasing the number of quarantined people. A negative consequence of increasing the adoption ratio is the increase in the number of false alerts, and thus, the number of people wrongly quarantined (curve $Q_S$ in Fig. 7b) due to the high value of the false positive ratio ($FPR$). If we improve the precision of digital contact tracing by reducing $FPR$ to 0.1 and increasing the true positive ratio $TPR$ to 0.8, we can see in Fig. 7c a significant reduction in the number of people quarantined. Particularly notable is the reduction in the number of false alerts. Increasing the precision also impacts slightly the number of infected people, which is reduced to 1.51 million.

**Figure 8:**
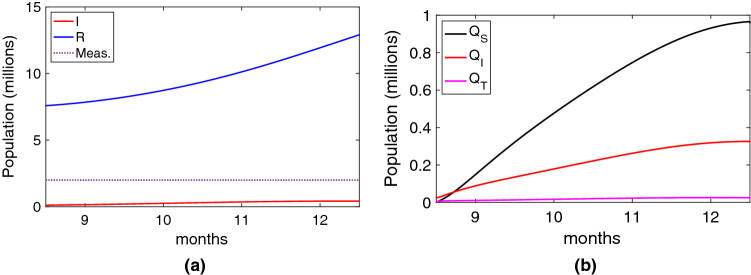
Contact tracing application efficiency considering that non stringent measures are taken and with excellent digital control tracing parameters: $$AR=0.7$$, $$ TPR=0.8 $$ and $$ FPR =0.1$$, $$TT=1$$. (**a**) Infected and recovered individuals; (**b**) People quarantined.

**Figure 9:**
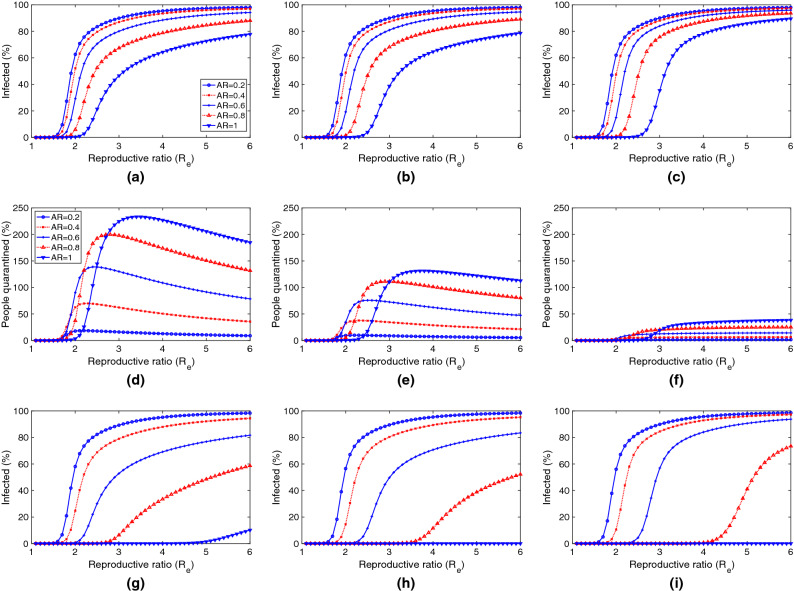
Future digital contact tracing efficiency depending on the reproductive ratio $$R_e$$ and for several adoption ratios, showing the percentage of the population infected and quarantined. Figures (**a**) and (**d**) show the results for a digital contact tracing approach with parameters similar to the ones deployed for the COVID-19. Figures (**b**) and (**e**) show the results when the accuracy is increased ($$ TPR $$ is increased (0.7 to 0.9), and $$ FPR $$ is reduced (0.4 to 0.2). Figures (**c**) and (**f**) show a full accuracy scenario with $$ TPR =1$$; The last row, figures (**g**), (**h**) and (**i**), show a centralised approach ($$TT=1$$, $$TC=1$$) with the same accuracy than figures (**a**), (**b**) and (**c**).

Now, in order to evaluate the impact of these factors on efficiency, we consider a scenario where no stringent measures are taken (as the one shown in Fig. 4c), so people are only quarantined when detected positive, or through digital contact tracing (no general lockdowns). This should be one of the goals of using digital contact tracing: to reduce the number of people unnecessarily quarantined. The results are shown in Fig. 8. We can see that, even with an increase of the adoption ratio to 0.7, an excellent precision ($$ TPR=0.8 $$ and $$ FPR =0.1$$), and with a contact tracing time of 1, the number of newly infected individuals in the considered period is still quite high (5.3 million).

Finally, we focus our study on the final number of infected and quarantined individuals as simple measures of the efficiency of digital contact tracing. We used a scenario similar to the previous one, where an outbreak of a future epidemic is produced with intensity $R_e$, and after six months of digital contact tracing we obtain the percentage of infected and quarantined people. Initially, ten individuals get infected, and the detection ratio is $\gamma =0.01$. We repeated this experiment depending on $R_e$, for different values of adoption ratio and considering several factors, such as the precision and the speed of contact tracing, which depend on the technology used. Note also that in the experiments, we have only plotted values between 1 and 6, as curves converge in this range.

The results are shown in Fig. 9. The first two Fig. 9a,d show the results for a digital contact tracing approach with parameters similar to the ones deployed for the COVID-19 ($$ TPR =0.7$$, $$ FPR =0.4$$, $$TT=2$$, $$TC=0.8$$). We can see that only when $$R_e$$ is lower than two and with high adoption ratios does the percentage of infected individuals remain low. In these cases, the proportion of people quarantined is not excessive, and thus digital contact tracing can be effective. Nevertheless, when $$R_e$$ is higher than 2, the infected and quarantined people are too high, so digital contact tracing is not applicable. Note that percentages greater than 100 mean that the same individual has been quarantined more than once or for periods longer than 14 days.

If we improve the accuracy by increasing $$ TPR $$ to 0.9, and decreasing $$ FRP $$ to 0.2, we can see that, although the infected individuals are slightly reduced (see Fig. 9b), the proportion of people quarantined is significantly reduced (the curves are slightly shifted to the right in Fig. 9e). This reflects a more selective (and effective) selection of individuals to quarantine. Finally, if we consider the utopian goal of no false positives and negatives, $$ TPR =1$$ and $$ FRP =0$$, we can see that the individuals quarantined are reduced to their minimum (Fig. 9f), reducing the number of infected people slightly. Summing up, improving accuracy has a significant impact on reducing the number of people quarantined.

Finally, we study the impact of having a centralised digital contact tracing solution, which relies mainly on the tracing time *TT* and the tracing coverage *TC*. The previous experiments considered a decentralised approach, with $TT=2$ days and $TC=0.8$. We repeated these experiments considering a centralised approach, with $TT=1$ and $TC=1$. The infected individuals are shown in Fig. 9g–i. Note that the results for the quarantine are not shown as they are very similar to Fig. 9a–c. Compared to the results obtained in the decentralised approach, we can clearly notice greater effectiveness at reducing the infected individuals, being particularly significant for higher adoption ratios (0.6 to 1). It is noteworthy that, with adoption ratios of 0.8 and 1, a centralised digital contact tracing can control outbreaks with $R_e$ of 3 or higher. Nevertheless, this is an unfeasible scenario (can only be considered for cohorts). If we consider more reasonable adoption ratios, that is, between 0.2 and 0.6, and the use of other measures that can reduce the effective reproductive ratio to values below 2, we can effectively reduce the number of infected individuals using a centralised digital contact tracing with greater accuracy. This is clearly noticeable when we can compare the infected individuals in Fig. 9h against the ones in Fig. 9a.

Summing up, although it is not directly a technical factor, we have seen that the critical factor impacting the overall effectiveness is the adoption ratio. As detailed in section "Digital contact tracing architecture", we can increase this adoption ratio, for example, with an opt-out app set up or by using it only for cohorts. Regarding technical factors, a centralised approach is clearly more effective than the decentralised one, reducing the number of infected individuals for the same value of $R_e$. Finally, increasing the accuracy has a huge impact on reducing the number of individuals quarantined.

## Discussion

The proposed model for evaluating the effectiveness and efficiency of digital control tracing has shown the main aspects that need to be improved for these applications’ current and future use.

Our model considers (as most epidemic models do) a homogenous population and contact distribution, which allows evaluating the dynamics of an epidemic in great populations in a speedy way. This is a shortcoming if we want to evaluate the particular spread of the disease depending on location, contact patterns, or age groups. To this end, we should consider the use of stochastic models or agent-based models, which would require a complete definition of the contact patterns and locations, and usually are computer-intensive based simulations. Nevertheless, as shown in the evaluation section for the English, Chinese and South Korean Apps, our model is a good approximation when considering a country’s population.

Although it is not a technical factor, the adoption ratio is the main factor that impacts the effectiveness of digital contact tracing. Only with high adoption ratios can an outbreak be controlled. Nevertheless, this is not a realistic scenario in most cases, and digital contact tracing must rely on (and complement) other measures to fight against an epidemic.

Regarding the efficiency of digital contact tracing, we have shown that improving accuracy on the estimation of risky contacts is a crucial issue, not only to reduce the quarantined people but also as a way to improve the people’s true in the application, thereby improving the adoption ratio. This accuracy is mainly a technical issue, which in the future can be improved with the combination of Bluetooth distance estimation with other sensing and machine learning techniques. Another key aspect is how the contacts are processed. A centralised approach will provide a faster tracing time by improving its efficiency. Nevertheless, it is necessary to ensure the required privacy requirements.

Summing up, digital contact tracing should be used as an add-on to standard epidemic mitigation measures, such as social distancing or manual contact tracing. This way, it can contribute to the reduction of cases while being more selective than general lockdowns.

## Conclusions

The key (and regretful) issue is why digital technology has failed to stem the worst pandemic in a century. The causes are not only technical; in fact, lack of coordination between countries and states, test shortages, and mistrust of technology are cited among the main social and political causes.

Summing up, based on the evaluations performed using our proposed model, we can derive the following conclusions:

- The efficacy of the deployed digital contact tracing applications, with an adoption ratio of around 20% (in countries where they were not mandatory), was quite limited. Therefore, it was necessary to use other measures.
- Adoption ratio is the critical factor in improving its effectiveness. Although it is not specifically a technical factor, this adoption ratio can be increased by installing and activating the app by default (known as the *opt-out* strategy) or by using it only for cohorts (subsets of the population).
- A higher accuracy in detecting the risky contacts is required to avoid false alerts and an excessive number of quarantined individuals. This accuracy plays a key role, as false alerts can undermine users’ confidence in digital contact tracing, thus reducing its adoption ratio.
- The implemented decentralised approach penalised the performance of digital contact tracing. Thus, a centralised approach provides faster contact tracing, which is essential to detect and quarantine possible newly infected individuals.
- Fortunately, with these technical improvements, and when combined with other mitigation measures, digital contact tracing can avert a reasonable number of infected individuals and deaths, even with adoption ratios around 20%, and high reproductive ratios.

## Data Availability

All the code used in the paper, including the models, is available on the following GitHub site: https://github.com/GRCDEV/DCT_EpiModel.
